# Persistent trigeminal artery in a patient with moyamoya disease:a case report and literature review

**DOI:** 10.1186/s12883-024-03545-y

**Published:** 2024-02-02

**Authors:** Tao Sun, Lixin Huang, Jun Sun, Zhimin Wu, Chuan Chen, Hui Wang

**Affiliations:** Department of Neurosurgery, Third Affiliated Hospital of Sun Yat-Sen University, No. 600th Tianhe Road, Guangzhou, 510630 Guangdong China

**Keywords:** Persistent trigeminal artery, Moyamoya disease, Computed tomography, Revascularization

## Abstract

**Backgrounds:**

Persistent trigeminal artery (PTA) is a rare anastomosis between internal carotid artery (ICA) and basilar artery. In rare conditions, the PTA could be combined with others cerebrovascular anomalies, moyamoya disease (MMD) is one of them.

**Case presentation:**

Here, we reported one rare case of MMD associated with PTA, the patient admitted to our department for severe dizziness and headache, imaging examination suggested MMD combined with right PTA, which arising from the ipsilateral cavernous portion of ICA. The patient received phased bilaterral revascularization with no any complication. In the subsequent follow-up, the patient’s symptoms and intracranial vascular condition gradually improved. Moreover, we conducted a literature review of coexistence of PTA and MMD, the results of a web of science regarding such condition, and a deep discussion providing brief insight into the status of co-occurrence of PTA and MMD, including its manifestation, treatment and outcome.

**Conclusions:**

The coexistence of PTA and MMD was rarely reported, the pathogenesis of such condition remains unknown. We found that the features of the coexistence of PTA and MMD were diverse, revascularization might be a feasible for such patient.

## Introduction

The persistent trigeminal artery (PTA) is the most common but rarely reported carotid-basilar anastomosis. Previous studies demonstrated that the incidence of PTA ranged from 0.1-0.6% [1], and the reports of PTA mainly focused on limited sample and case reports. The presence of PTA could combine with many other diseases, such as aneurysm [2–4], arteriovenous malformation [5, 6], and trigeminal neuralgia [7–9]. Moreover, moyamoya disease (MMD) is a rare disease characterized by occlusion and abnormal proliferation of the cerebral arteries and the formation of moyamoya vessels, which could ultimately lead to the occurrence of cerebral infarction or hemorrhage [10]. The revascularization, which could greatly reduce the incidence of hemorrhage and cerebral infarction, has been proved to be a safe and effective choice for patient with MMD after extensive clinical studies and practices. In very condition, the MMD could combine with PTA, no matter whether this artery is affected [2, 11]. To our knowledge, only 13 cases of MMD associated with PTA were reported in the past decades (including current case).

## Case presentation

**Fig. 1:**
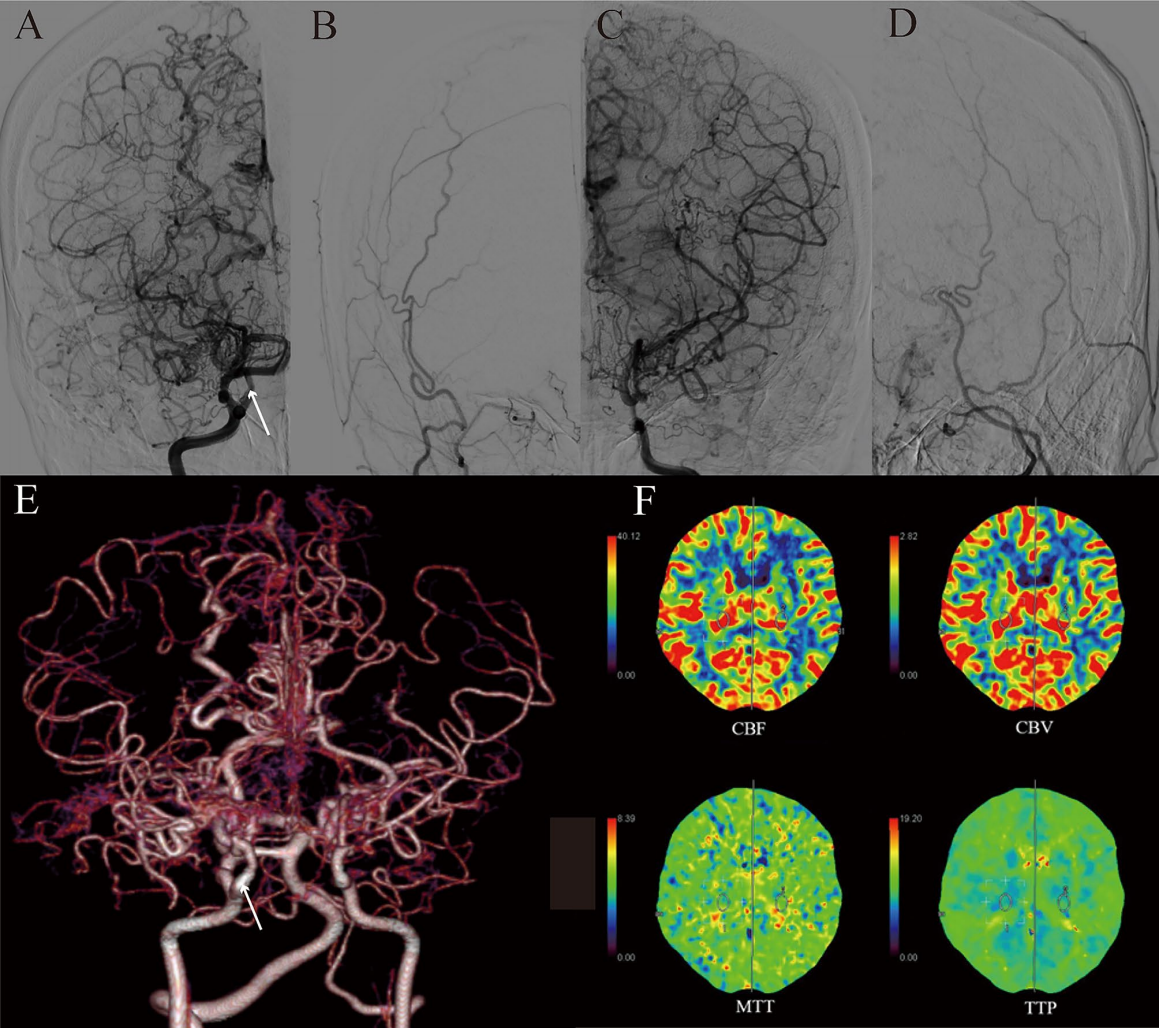
First admission CTP imaging showed old infarction and partial liquefaction in the left frontal and temporal lobe (**F**), and the CTA and DSA revealed the PTA arises from cavernous portion of the right ICA (**A, E**) (white arrow), and stenosis or occlusion in the bilateral ACAs, the M1 segments of bilateral MCAs, and the C5-C6 segments of bilateal ICAs, but the anterior and posterior communicating arteries were patent (**A, B, C, D, E**). Moreover, there were a great mount of moyamoya vessels (**A, B, C, D, E**). ICA: internal carotid artery; CTP: computed tomography perfusion; CTA: computed tomography angiography; DSA: digital subtraction angiography. ACA: anterior cerebral artery; MCA: middle cerebral artery; PTA: persistent trigeminal artery

**Fig. 2:**
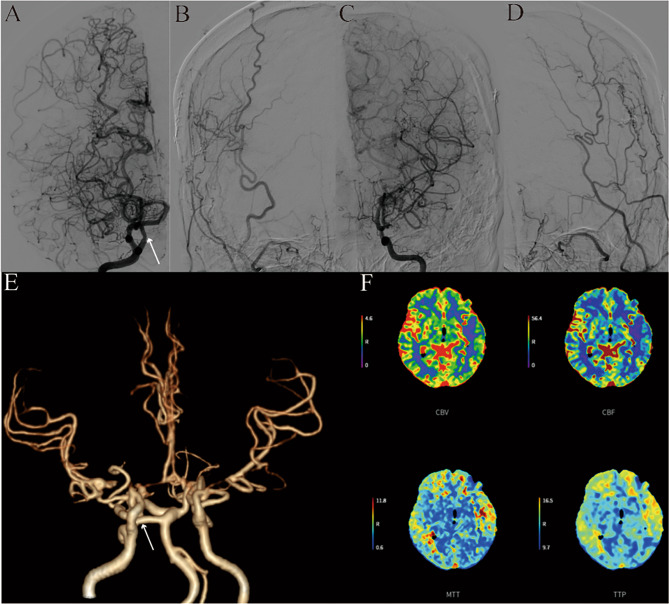
The latest imaging test and DSA (more than two years later after first admission) showed the formation of numerous collateral vessels at the site of bilateral indirect (**B, D**) and a significant improvement in intracranial blood flow conditions (**A, B, C, D**) compared to first admission, but there was low cerebral blood flow perfusion in the left frontal and temporal lobe (**F**)

The case involved a 44-year-old woman who admitted to our department for further treatment. She experienced sudden, severe dizziness and headache three months ago without any identifiable cause. The head computed tomography scan performed at local hospital revealed infarction in the left temporal and basal ganglia, as well as severe stenosis of both internal carotid arteries (ICAs). She was initially treated symptomatically and gradually improved. Three months later, she scheduled for further treatment in our department. The computed tomography perfusion (CTP) imaging showed old infarction and partial liquefaction in the left frontal and temporal lobe. Additionally, the computed tomography angiography (CTA) and digital subtraction angiography (DSA) revealed a PTA arising from cavernous portion of the right ICA, and stenosis or occlusion in the bilateral anterior cerebral arteries (ACA), the M1 segment of bilateral middle cerebral arteries (MCAs), and the C5-C6 segment of bilateral ICAs, combining with many net-like vessels, but the anterior and posterior communicating arteries were patent (Fig. 1). Following thorough preoperative examinations, it was determined that bilateral revascularizations were needed. Considering the characteristics of the disease onset, the left revascularization was performed first. During the surgery, however, we found that all of the potential recipient vessels had a diameter smaller than 0.5 mm, while the superficial temporal artery had a diameter greater than 1.5 mm, a direct bypass would pose a high risk of postoperative hemorrhage. Therefore, an indirect bypass procedure was performed on the left side, the patient recovered well and was discharged within a week. During the follow-up visit three months later, imaging examination revealed severe stenosis of the arteries. However, there were improvements in the intracranial vascular conditions, and collateral vessels had formed on the left side. For similar reason mentioned above, an indirect bypass on the right side was performed during this hospitalization. The patient experienced a smooth recovery and symptom improvement. The most recent CTA, CTP tests and DSA of the head indicated significant improvement in both intracranial and extracranial collateral vessels. However, there was still relatively low cerebral blood flow perfusion in the left frontal and temporal lobe (Fig. 2).

## Discussion

**Fig. 3:**
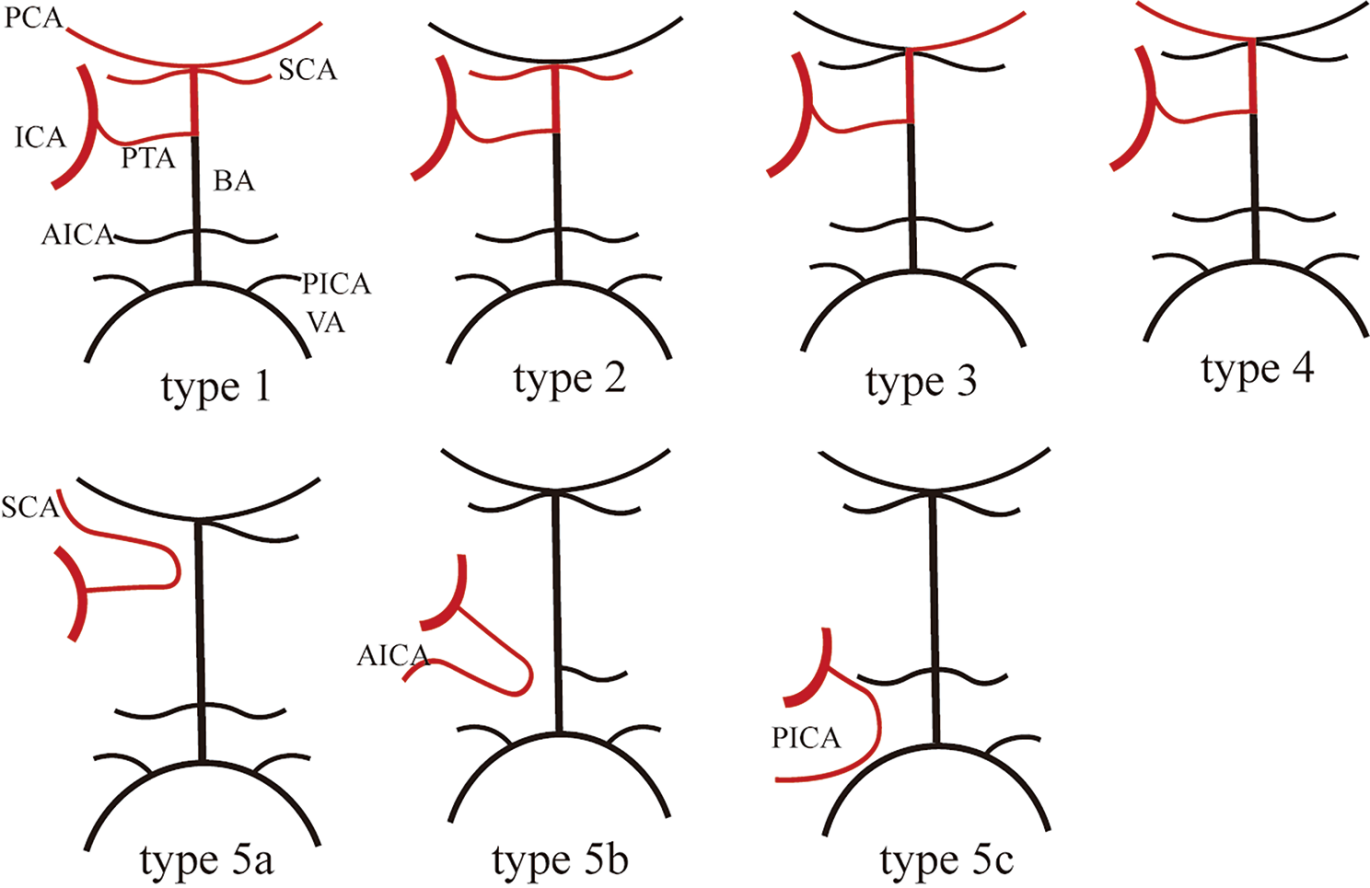
Weon subtyped the PTA into five types based on its hemodynamic characteristics: type 1, the PTA supplies bilateral PCA, and superior cerebellar artery; type 2, the PTA supplies of the bilateral superior cerebellar artery; type 3, the PTA supplies the contralateral PCA; type 4, the PTA supplies ipsilateral PCA; and type 5 can be subclassified as **5a**,**5b**, and **5c** according to the terminative artery, the superior cerebellar artery (**5a**), anterior inferior cerebellar artery (**5b**), and the posterior inferior cerebellar artery (**5c**), respectively. PTA: persistent trigeminal artery; ICA: internal carotid artery; SCA: superior cerebellar arteries; AICA: anterior inferior cerebellar artery; PICA: posterior inferior cerebellar artery; BA: basilar artery; VA: vertebral artery; PCA: posterior cerebral artery

PTA, otic artery, hypoglossal artery, and proatlantic intersegmental artery are the four persistent embryologic carotid-basilar anastomoses, and PTA is the most common with a very low reported incidence [1]. It was first described in a specimen in 1841 [12] and demonstrated in a live subject in 1950 [13]. Based on its radiological and hemodynamic characteristics, Saltzman classified PTA into three types [14]. Type 1 is the most common, the PTA supplies the bilateral superior cerebellar arteries (SCAs) and the bilateral posterior cerebral arteries. In type 2, the PTA supplies bilateral SCAs. Type 3 represents a variant of PTA, which can be further subdivided into three subtypes: type 3a, where PTA directly drains into the ipsilateral SCA; type 3b, where PTA directly drains into the ipsilateral anterior inferior cerebellar artery (AICA); and type 3c, where PTA directly drains into the ipsilateral posterior inferior cerebellar artery (PICA). Salas classified PTA into lateral and medial types based on the anatomical relationship between PTA and the abducent nerve [15]. Weon further expanded the classification into five types [16]. Types 1 and 2 correspond to Saltzman’s classification, while in types 3 and 4, the PTA drains into the contralateral and ipsilateral posterior cerebral arteries, respectively. Type 5 can be further divided into subtypes based on its terminal arteries: type 5a (SCA), type 5b (AICA), and type 5c (PICA). (Fig. 3). It was reported that the type 1 was the most common [17].

**Fig. 4:**
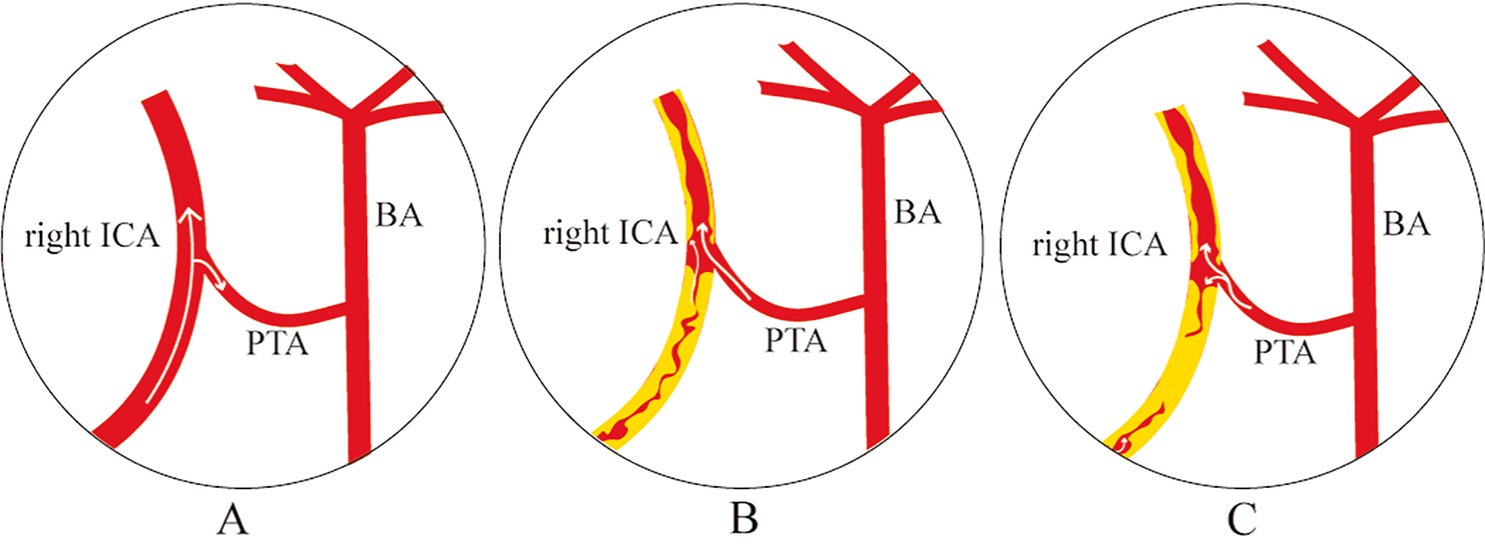
The figure illustrates the role of PTA in patient with MMD. Under normal blood flow, the ICA supplies blood to the BA via PTA (**A**). However, in patients with MMD, the ICA get stenosis (**B**) and even occlusion (**C**), resulting in the flow of blood from BA to ICA via PTA partially (**B**) and totally (**C**). PTA: persistent trigeminal artery; MMD: moyamoya disease; ICA: internal carotid artery; BA: basilar artery

The PTA draws attention for it’s usually reported to be associated with central nervous system disease, such as aneurysm [2–4], arteriovenous malformation [5, 6], MMD [2, 11], and trigeminal neuralgia [7–9]. It’s reported that the rough incidence of MMD with PTA is 0.0021% [11]. After carefully retrieval in web of science, we found that there were only 13 cases of patients with the coexistence of PTA with MMD (including current case). (Table 1) For the vascular features of MMD and PTA, the PTA could be the collateral vessel of MMD and protect the patients from complications, such as ischemia and hemorrhage (Fig. 4), but also prompt the stage of MMD [18]. Such patients mostly present with ischemia, with a small part with hemorrhage, and no age, gender, or lateralization patterns difference. Patients who underwent revascularization procedures generally achieved favorable outcomes. As the PTA coexistence with MMD is rarely reported, the incidence, clinical feature, and outcome of such condition remain further study.

In addition to PTA, the MMD could be combined with other anomalies, such as arteriovenous malformation [19–21], carotid artery absence [22–24], ICA hyperplasia [25] and so on. It is more likely that the increased blood to the arteriovenous malformation and increased blood turbulence lead to intimal hyperplasia and progressive occlusion [26]. The anastomotic vessels and their capillaries could open up and become distended in the presence of ischemia, these capillaries could only discharge the increased blood into normal veins which in tum become distended [19]. As the variant of PTA is usually poorly visualized, more attentions should be paid to such patient [25]. In patients with carotid artery abnormality, the presence of PTA could be vital collateral circulation [22, 23]. There is no currently direct etiological evidence show the relationships between MMD and these diseases, but it can be speculated that congenital factors may play an important role [22], as the timeframe in which the PTA regresses during embryonic development coincides with the period in which the vascular condition bears resemblance [27]. Uchino reported the higher incidence of PTA in patient also support this hypothesis [28], but the potential mechanism remains unknown [29]. As the imaging characteristics of MMD, the PTA could be the solely blood supply to the affected brain region [18]. Moreover, this artery could even change the haemodynamics of patients with MMD, and be used as a route for endovascular coiling for aneurysm [2]. Also, the PTA could play a protective role in cases of acquired occlusion of the ipsilateral ICA [30, 31], it could provide compensatory blood to the targeted area. In currtent case, the proximal basilar artery and bilateral vertebral arteries were very thin, the PTA could supply blood to the posterior circulation, especially in patient with MMD, whose cerebral arteries were badly affected. However, further studies are still needed to explore their connections.

**Table 1:** Summary of the 13 cases of patients with the coexistence of PTA and MMD

Reference	Year	Gender	Age(Y)	Complaints	Side	Imaging finding on admission	Treatment (L/R)	Outcome
Handa, J [32].	1972	/	/	/	right	MMD and PTA	/	/
Chen, S. T [27].	1993	F	64	left caudate and intraventricular hemorrhage	bilateral	MMD, bilateral PTA and aneurysm	/	/
Kwak, R [11]	1983	M	44	left temporoparietal lobe hematoma	right	MMD, PTA and aneurysm	/	not difficult
Kwak, R [11]	1983	F	56	right temporoparietal lobe hematoma	left	MMD and PTA	/	normal
Otsuki, T [33].	1982	M	51	/	/	/	/	/
Kinjo, T [18].	1988	F	16	right hemiparesis	right	MMD and PTA		
Uchino, A [28].	2002	M	9	transient left hemiparesis	left	MMD and PTA	right bypass	uneventful
Komiyama, M [34].	1998	M	3	sudden weakness of right upper extremity	left	MMD and PTA	bilateral bypass	not favorable
Hou, K [2].	2019	M	56	headache, nausea, vomiting	left	MMD, PTA and multiple aneurysms	/	/
Tan, E. C [35].	1991	M	35	suddenly lost consciousness	right	MMD and PTA	bilateral bypass	uneventful
Suzuki, S [29].	1996	F	6	right hemiparetic gait	left	MMD and PTA	bilateral bypass	normal
Suzuki, S [29].	1996	F	2	left hemiparesis	left	MMD and PTA	bilateral bypass	normal
Current case	2023	F	44	sudden and severe dizziness, headache	right	MMD and PTA	bilateral bypass	normal

Abbreviation: M: male; F: female; Y: year; L: left; R: right; MMD: moyamoya disease; PTA: persistent trigeminal artery

## Conclusions

The coexistence of PTA and MMD was rare reported, the pathogenesis of such condition remains yet to be explored. Based on the literature review, we found that the manifestation of such patient showed no classical characteristics. Revascularization might be a feasible choice for patient with PTA combined with MMD.

## Data Availability

Not applicable.

## Abbreviations

PTA: Persistent trigeminal artery
ICA: Internal carotid artery
MMD: Moyamoya disease
ACA: Anterior cerebral artery
MCA: Middle cerebral artery
CTP: Computed tomography perfusion
CTA: Computed tomography angiography
SCA: Superior cerebellar arteries
AICA: Anterior inferior cerebellar artery
PICA: Posterior inferior cerebellar artery
BA: Basilar artery
VA: Vertebral artery
PCA: Posterior cerebral artery
DSA: Digital subtraction angiography
